# Cellular senescence as a key contributor to secondary neurodegeneration in traumatic brain injury and stroke

**DOI:** 10.1186/s40035-024-00457-2

**Published:** 2024-12-12

**Authors:** Zhihai Huang, Peisheng Xu, David C. Hess, Quanguang Zhang

**Affiliations:** 1 Department of Neurology, Medical College of Georgia, Augusta University, 1120 15th Street, Augusta, GA 30912 USA; 2 Department of Pharmacology, Toxicology and Neuroscience, Louisiana State University Health Sciences Center, 1501 Kings Highway, Shreveport, LA 71103 USA; 3 Department of Drug Discovery and Biomedical Sciences, College of Pharmacy, University of South Carolina, 715 Sumter, Columbia, SC 29208 USA

**Keywords:** Traumatic brain injury, Stroke, Neurodegeneration, Cellular senescence, Senolytic therapy

## Abstract

Traumatic brain injury (TBI) and stroke pose major health challenges, impacting millions of individuals globally. Once considered solely acute events, these neurological conditions are now recognized as enduring pathological processes with long-term consequences, including an increased susceptibility to neurodegeneration. However, effective strategies to counteract their devastating consequences are still lacking. Cellular senescence, marked by irreversible cell-cycle arrest, is emerging as a crucial factor in various neurodegenerative diseases. Recent research further reveals that cellular senescence may be a potential driver for secondary neurodegeneration following brain injury. Herein, we synthesize emerging evidence that TBI and stroke drive the accumulation of senescent cells in the brain. The rationale for targeting senescent cells as a therapeutic approach to combat neurodegeneration following TBI/stroke is outlined. From a translational perspective, we emphasize current knowledge and future directions of senolytic therapy for these neurological conditions.

## Background

Acquired brain injury, including traumatic brain injury (TBI) and stroke, represents a major cause of disability and mortality in adults [1]. Globally, millions of people suffer from TBI and stroke annually, imposing substantial burdens on both patients and society [2, 3]. Although TBI and stroke arise from different primary insults, a growing body of evidence highlights their shared pathophysiological features, including tissue destruction, excitotoxicity, oxidative stress, apoptosis, inflammation, and compromised cellular integrity, culminating in neuronal death [4, 5]. This overlap suggests that therapeutic strategies targeting these common pathological mechanisms could confer benefits for both TBI and stroke [4]. Rather than acute central nervous system (CNS) insults, these neurological conditions are increasingly recognized as chronic health issues [6, 7]. Progress has also been made in improving the acute care of these neurological conditions. However, effective treatments to improve long-term prognosis remain a challenge [8, 9]. Many TBI/stroke survivors endure long-term disability, including cognitive impairment and depression [10–12]. Epidemiological studies also revealed a positive correlation between a history of TBI/stroke and an increased risk of developing neurodegenerative diseases including Parkinson’s disease (PD), Alzheimer’s disease (AD), and other dementias [13–16]. Indeed, TBI and stroke are increasingly recognized as modifiable risk factors for neurodegenerative diseases. Thus, the development of strategies to target secondary neurodegeneration following these neurological conditions is urgently warranted.

Cellular senescence is considered a state of irreversible cell cycle arrest [17]. In normal physiological processes, cellular senescence engages in embryonic development and tissue remodeling [18]. Nevertheless, under pathological conditions, cellular senescence could also lead to a proinflammatory milieu, impeding the replication of damaged cells and compromising cellular function [18, 19]. Research has shown enhanced senescence phenotype and biomarkers in both experimental animal models and patients with a history of TBI/stroke [20, 21]. Preliminary experimental evidence also reveals the potential of senolytic therapies in combating secondary neurodegeneration after brain injury [22].

Here, we describe cellular senescence as a potential trigger for secondary neurodegenerative disorders after TBI and stroke. The present and the future of senolytic therapy for these neurological conditions are also discussed. We propose that elimination of senescent cells may represent an attractive target to limit neurodegeneration resulting from TBI/stroke.

## TBI, stroke, and secondary neurodegeneration

Increasing evidence has shown that TBI and stroke may trigger the progression of neurodegeneration and elevate the risk of developing diseases such as PD, AD, and other dementias [13–15, 23]. Retrospective cohort studies have shown that athletes engaged in collision sports are at a higher risk of developing neurodegenerative diseases [24, 25]. In one such study involving 7676 former professional soccer players and 23,028 controls from the general population, researchers observed a significantly increased risk of developing neurodegenerative diseases, including AD and PD, among soccer players [24]. These athletes also had a higher mortality rate from neurodegenerative diseases and were prescribed dementia-related medications more frequently than the controls [24]. A study examined the relationship between TBI history and the risk of AD and related dementias. Results demonstrated a positive correlation between TBI history and an increased risk of developing AD and related dementias, which was observed across all severities of TBI [26]. These findings were further supported by a meta-analysis that showed an increased risk of dementia among individuals with TBI [15]. A nationwide cohort study also reported an increased risk of PD among military service veterans, particularly those who had experienced TBI or post-traumatic stress disorder [27].

Likewise, a meta-analysis of 48 studies involving over 3 million participants found that stroke is an independent and potentially modifiable risk factor for dementia [23]. There is a strong association between a history of stroke, including both prevalent and incident strokes, and an increased risk of all-cause dementia [23]. In a cohort study of 246,686 women diagnosed with breast cancer, the incidence of AD and related dementias was found to be higher in women who had a stroke (40.24%) than in those who did not (31.34%) [28]. In patients with both familial and sporadic AD, a history of stroke is also significantly associated with an increased disease risk [29]. Proteomic analyses revealed the aggregation of proteins related to neurodegeneration, such as TDP43, FUS, hnRNPA1, and PSF/SFPQ, following experimental cerebral ischemia [30]. These results highlight the possible role of ischemic events in accelerating neurodegenerative processes.

Indeed, TBI and stroke can trigger neuronal damage and tissue loss in both perilesional regions and remote regions, which has been referred to as secondary neurodegeneration [31, 32]. Overt neuropathologies, such as loss of myelinated fibers and axonal swellings, have been reported in veterans with a history of TBI [33]. Repetitive head injury has been associated with a unique neurological deterioration known as chronic traumatic encephalopathy (CTE) [34]. CTE is a neurodegenerative disease characterized by hyperphosphorylated tau pathology, multifocal axonal varicosities, and axonal loss [35–37]. Unlike the tau pathology seen in other neurodegenerative disorders, hyperphosphorylated tau in CTE usually appears in a patchy manner, distributed around perivascular areas [38]. Post-mortem studies have revealed a significantly increased risk of CTE in college and professional football players who have experienced repeated head injuries [35]. Among individuals neuropathologically diagnosed with CTE, 85% of those with mild CTE reported cognitive symptoms, and 33% had signs of dementia before death [35]. For severe CTE patients, 95% reported cognitive symptoms, and 85% had signs of dementia [35].

Convergent evidence from both experimental animal and human studies suggests that TBI/stroke may trigger aberrant accumulation and deposition of amyloid-beta (Aβ) in the brain. Post-mortem studies revealed that, compared to uninjured age-matched controls, patients with a history of a single TBI exhibited widespread neurofibrillary tangles and Aβ plaques in their brains [39]. In individuals who died from TBI, extensive deposits of amyloid precursor protein (APP), Aβ, and neurofilament protein were found in swollen axons [40]. Likewise, AD-like Aβ deposition was observed in patients who experienced stroke/transient ischemic attack, which was associated with a more severe and rapid cognitive decline within three years after the stroke [41]. Findings from experimental animal models revealed that exposure to ischemia increases the activities of β- and γ-secretases, enzymes involved in the processing of APP and amyloidogenic pathways [42–44]. Conversely, it decreases the activity of α-secretase, an enzyme responsible for non-amyloidogenic pathways in APP processing [42].

It is noteworthy that TBI/stroke shares several pathological features with neurodegenerative diseases, with cellular senescence emerging as a central aspect. As a key player in neuronal degeneration, Aβ oligomers could directly accelerate senescence in neuronal cells. Exposure of cultured mouse hippocampal neural stem/progenitor cells to Aβ42 oligomers triggers senescence, impeding cell proliferation and differentiation [45]. Similar results were observed in primary mouse neurons treated with Aβ42 [46]. Interestingly, senescent oligodendrocyte progenitor cells have been found surrounding Aβ plaques in both AD patients and a mouse model of AD, while elimination of these senescent cells attenuates AD-associated cognitive deficits [47]. A post-mortem study also revealed an increased proportion of senescent neurons in the brains of AD patients [48]. This suggests the potential to target senescent cells to combat the progressive neuropathology following TBI/stroke. In the subsequent sections, we discuss the role of cellular senescence in these neurological conditions and how it could be a therapeutic target.

## Cellular senescence as a driver for chronic inflammation and neurodegeneration

Cellular senescence is thought to be a permanent state of cell cycle arrest with both beneficial and detrimental implications [18]. Triggered by various damaging stimuli such as oncogenes, telomere attrition, reactive oxygen species (ROS), DNA damage response, and mitochondrial dysfunction, this distinctive cellular phenotype has been extensively reviewed [17, 18]. Importantly, senescent cells could acquire a complex response known as senescence-associated secretory phenotype (SASP), which involves the secretion of pro-inflammatory chemokines, cytokines, and growth factors [17, 49]. As an inflammation signal, SASP recruits immune cells to the sites of pathology, thereby promoting the removal of senescent cells [17, 50]. This "senescence, clearance, followed by regeneration" process aids in wound healing, tissue regeneration, and the mitigation of tissue fibrosis [17, 50].

In pathological contexts, nevertheless, compromised clearance mechanisms may result in the accumulation of senescent cells [51, 52]. Unchecked SASP may extend its influence to neighboring cells and, in some cases, transform normal cells into senescent ones through a phenomenon known as paracrine senescence [53]. Mediators involved in this paracrine action include ROS, chemokines, and more recently identified small extracellular vesicles [53–55]. The persistence of these senescent cells within tissues blunts the replenishment of functional cells, ultimately diminishing tissue regenerative potential and function. Eliminating senescent cells, therefore, is critical for the maintenance of normal cellular function and tissue homeostasis in such cases.

Although senescent cells typically account for only 4%–15% of the total cell population, they are increasingly recognized as major contributors to chronic inflammation [56–58]. These cells accumulate in multiple tissues with aging, and the clearance of p16^INK4A^-positive senescent cells has been shown to significantly reduce the expression of proinflammatory mediators across various tissues [59]. In line with this evidence, selective elimination of senescent cells using a senolytic cocktail has been demonstrated to alleviate the secretion of proinflammatory cytokines in aged mice and human adipose tissue [60]. Whole-body senescent cell clearance has been shown to alleviate age-related brain inflammation, microglial activation, and the infiltration of immune cells [61]. Conversely, transferring blood from aged mice to young mice results in increased systemic inflammation in the young mice [62]. This effect is eliminated when the aged mice are treated with senolytic drugs prior to the blood exchange [62]. These findings collectively point to the critical role of cellular senescence in the initiation and maintenance of inflammation.

Indeed, chronic low-level inflammation is increasingly recognized as a key factor in the onset of progressive neurodegeneration. Studies have shown that a single systemic administration of lipopolysaccharide or TNFα can activate microglia and increase the expression of pro-inflammatory factors in the brain, leading to chronic inflammation and neuronal loss, particularly in the substantia nigra [63]. Another study shows that lipopolysaccharide-mediated inflammation results in neuronal degeneration in the cerebral cortex and hippocampal CA1 and CA3 regions. These detrimental effects can be mitigated by reducing the inflammatory response [64]. The activation and assembly of the NLRP3 inflammasome lead to significant neuronal death in a microglia-neuron co-culture model [65]. In line with these findings, genetic or pharmacological inhibition of the NLRP3 inflammasome or IL-1β diminishes the rise of pro-inflammatory mediators induced by lipopolysaccharides and mitigates neuronal degeneration progression [66].

It is noteworthy that low-level chronic inflammation is a common feature of various neurodegenerative diseases including AD and PD [67–69]. A meta-analysis involving 2629 patients and 2049 healthy controls has revealed that certain pro-inflammatory cytokines are significantly elevated in the cerebrospinal fluid (CSF) of individuals with AD and PD [67]. Specifically, levels of TGF-β, MCP-1, and YKL-40 are increased in the CSF of AD patients while patients with PD show elevated levels of TGF-β1, IL-6, and IL-1β in their CSF [67]. Similarly, high levels of senescence markers are commonly observed in patients with neurodegenerative diseases [70–72]. Alleviating inflammation or eliminating senescent cells has been shown to counteract these degenerative processes significantly [73, 74]. Genetic deletion of the NLRP3 inflammasome reduces tau propagation and hippocampal atrophy in a tauopathy mouse model [75]. Consistent results have been found in another study, where mice deficient in the NLRP3 inflammasome display reduced tau hyperphosphorylation following hippocampal infusion with brain homogenate from transgenic AD mice [73]. Along the same lines, selective removal of senescent cells notably decreases the burden of tau-containing neurofibrillary tangle, loss of neurons, and cerebral ventricular enlargement [76]. Cellular senescence, therefore, might serve as a critical intersection between chronic inflammation and neurodegeneration.

## Biomarkers to identify senescent cells

Several biomarkers for senescent cells have been proposed. Senescence-associated β-galactosidase (SA-β-Gal) is one of the most widely used biomarkers [77, 78]. Unlike typical lysosomal β-Gal, which exhibits peak activity at a pH 4.0 to 4.5, SA-β-Gal is detectable at pH 6 due to increased lysosomal enzyme levels and its residual activity in senescent cells [77, 79]. This unique characteristic makes it a robust and sensitive approach for identifying senescent cells, as its activity is confined to these cells and is largely absent in non-senescent cells.

Since cellular senescence is characterized by cell cycle arrest, increased levels of cyclin-dependent kinase inhibitors—such as p16^INK4A^, p53, and p21—are recognized as defining hallmarks of senescent cells [80, 81]. Elevated p16^INK4A^, typically associated with replicative senescence, is highly expressed in most senescent cells [52, 59]. Meanwhile, p53, a transcription factor activated by cellular stress, especially DNA damage response and oxidative stress, can induce the expression of p21. The p53/p21 pathway is believed to be one of the early activators in the senescence process [81, 82]. In addition, as discussed in the previous section, senescent cells secrete a variety of SASP factors, providing an indirect means of assessing the senescent state. The profile of the SASP, nevertheless, is highly heterogeneous across different senescent cell types [83]. This inherent variability might limit the direct application of SASP profiling for detecting senescence. Senescent cells also exhibit distinct morphological changes, as discussed in a previous review [84]. The loss of the nuclear lamina protein lamin B1, commonly observed in senescent cells across various contexts, has been proposed as a hallmark of cellular senescence [85, 86].

Despite the discovery of these biomarkers, it is crucial to recognize that no single hallmark can accurately identify senescent cells due to their heterogeneous nature [82, 87]. Not all these senescent biomarkers are expressed in every senescent phenotype, and some of these traits may also be observed during certain physiological processes. It has been proposed that three distinct features are required to accurately identify senescent cells: cell cycle arrest, structural changes, and additional senescence markers such as DNA damage, elevated ROS levels, and specific SASP factors [82]. A comprehensive assessment of multiple senescence-associated traits, therefore, is necessary for reliable detection.

## Cellular senescence in TBI and stroke: friend or foe?

Several studies have documented widespread cellular senescence following TBI and stroke. In a mouse model of TBI, senescent neurons, astrocytes, and microglia were detected as early as 7 days post-injury [21]. These abnormalities worsened over time, with more pronounced cellular senescence observed months after the initial injury [22, 88]. Concurrent with senescent cells, elevated levels of DNA damage biomarkers were reported, indicating that DNA damage might act as a trigger for TBI-induced cellular senescence [89–91]. Other pathological changes resulting from TBI, such as ROS generation and mitochondrial dysfunction, may also contribute to TBI-triggered senescence, although direct evidence is still lacking [92, 93]. A post-mortem study provided additional insights into cellular senescence in TBI [20]. This study, involving a case series of 38 professional athletes with a history of repeated mild TBI, revealed increased DNA damage and expression of cellular senescence and SASP pathways in their brains [20]. Additionally, abnormal neuron morphologies were observed, including the loss of emerin, a structural integral protein in the nuclear membrane, alongside reduced expression of myelin basic protein and DNA repair proteins [20].

Similarly, in rodent models of ischemic stroke, elevated levels of senescence markers, including p16^INK4A^ and p21, were observed in the infarct area 72 h after transient middle cerebral artery occlusion (MCAO) and photothrombotic stroke, two widely used rodent models of ischemic stroke [94, 95]. Further post-mortem analysis of brain samples from a patient with ischemic stroke revealed a higher number of p16^INK4A^-positive cells in the perimeter of the infarct area compared to other areas [94]. However, this was a small-scale exploratory study with a limited sample size, and further research with larger patient samples is required to verify this finding. Also, mRNA levels of senescence markers were found to increase as early as 12 h post-MCAO, particularly in astrocytes and endothelial cells [96]. Similar results have been observed in oxygen–glucose deprivation-treated astrocytes and endothelial cells, an ischemia-like in vitro model [96]. These findings suggest a potential role of cellular senescence in TBI and stroke.

While senescent cells are extensively observed in both early and late stages following TBI and stroke, their precise role in the different phases remains unclear. Indeed, emerging evidence suggests that cellular senescence triggered by injuries may not solely be pathogenic but could also signal tissue repair. For instance, in zebrafish, cellular senescence occurs at the site of damage following pectoral fin amputation, and the removal of these senescent cells during the early stages impedes fin regeneration [97]. Similarly, systemic deletion of senescent cells in young mice hampers muscle regeneration following injury [98]. This suggests that "early senescence" might actively participate in the tissue repair process. However, there has been no comprehensive investigation into the potential differing roles of senescent cells during different phases following TBI or stroke.

Using an ultrasensitive reporter to detect biomarkers of cellular senescence, a recent study observed senescent cells within young and healthy tissues [99]. These senescent cells play a critical role in tissue regeneration by sensing tissue inflammation and secreting SASP. Eliminating these senescent cells significantly diminishes the recruitment of resident stem cells and epithelial regeneration [99].

**Fig. 1:**
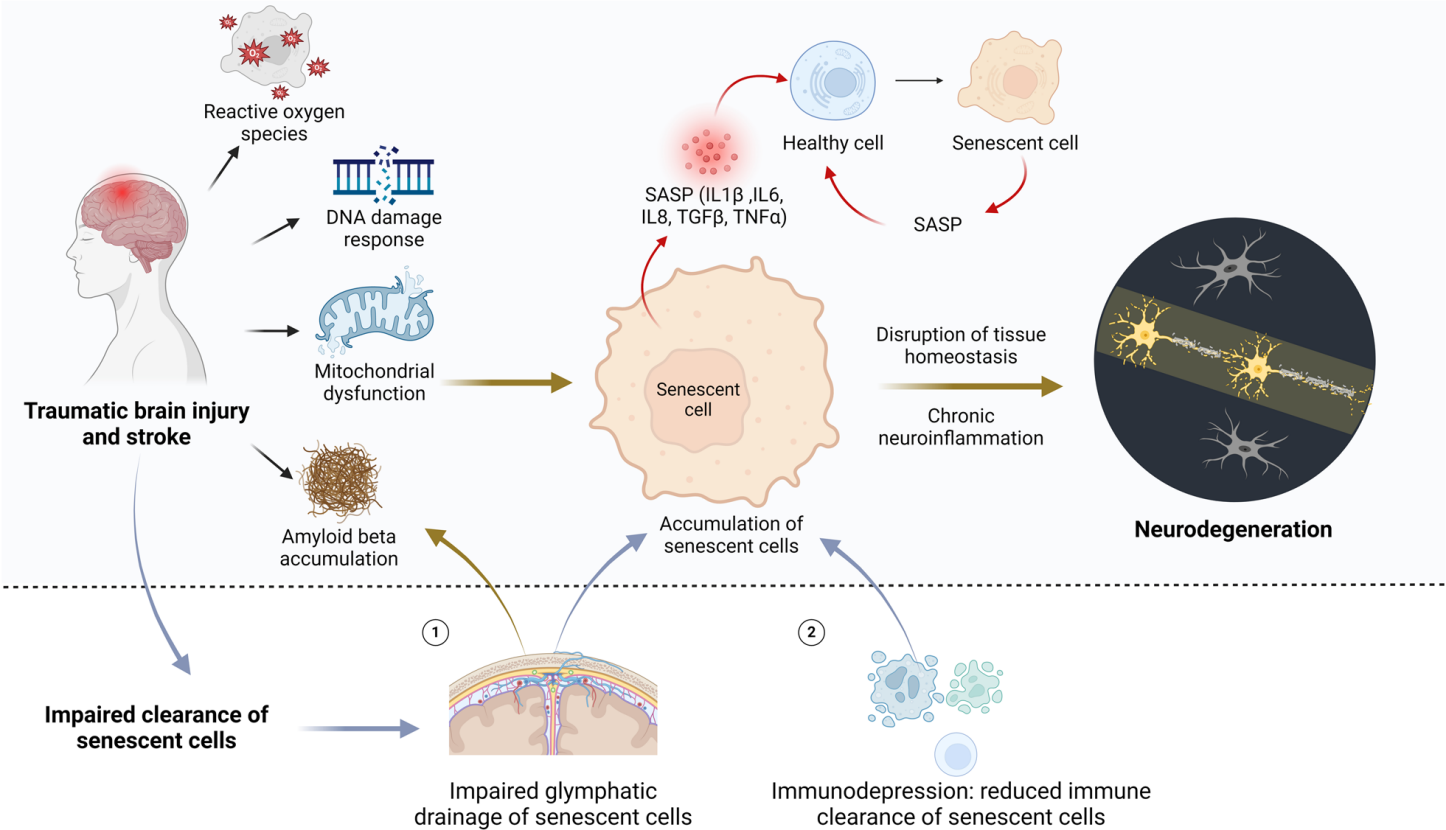
Proposed mechanisms underlying cellular senescence following TBI and stroke. DNA damage response, ROS, and mitochondrial dysfunction resulting from TBI may initiate cellular senescence in the brain. These senescent cells subsequently induce SASP, potentially transforming healthy cells into senescent ones. Furthermore, TBI/stroke may impair glymphatic drainage function and induce immunodepression, exacerbating the accumulation of senescent cells in the brain. The accumulation of senescent cells disrupts tissue homeostasis, leading to chronic neuroinflammation, ultimately culminating in neurodegeneration. Abbreviations: SASP, Senescence-associated secretory phenotype; TBI, Traumatic brain injury; ROS, Reactive oxygen species

Preliminary evidence suggests that senolytics, when administered 24 h after cerebral ischemia, may yield beneficial effects [96, 100]. However, since senescent cells can be observed just hours after cerebral ischemia, the role of very early senescent signaling in this condition remains underexplored. Additionally, the role of "early senescence" in TBI is still largely unknown. Given this context, it is plausible to speculate that "early senescence" in TBI and stroke may also act as an endogenous signal to facilitate the removal of pathological cells and promote tissue regeneration, which remains to be further verified. Such research could enhance our understanding of senescence in these conditions and reveal new opportunities for therapeutic interventions. Figure 1 illustrates TBI/stroke-induced cellular senescence and the proposed mechanisms involved.

## TBI, stroke, and impaired clearance of senescent cells

Although TBI and stroke can directly induce cellular senescence through various mechanisms, the impaired removal of senescent cells may further exacerbate their accumulation in the brain. Physiologically, immune cells such as T cells and macrophages play crucial roles in recognizing and clearing senescent cells [101–103]. Immunosenescence, the deterioration of the immune system due to aging, and impaired immune surveillance, can result in the exacerbated accumulation of diverse senescent cells [51, 104]. The attrition and senescence of immune cell populations lead to compromised immune function, which in turn can trigger further senescence, an effect that can be counteracted by the transplantation of young immune cells [104].

Intriguingly, although the exact mechanism is not fully understood, brain injury, including TBI and stroke, can induce immunodepression characterized by systemic downregulation of innate and adaptive immunity [105–108]. Early studies have reported a significant decrease of T-cell function and/or activation to below normal levels within months after CNS injury [109, 110]. This suppression of cellular immunity and the decrease in helper T cells have also been observed in patients suffering from severe head injury [111]. The term “CNS injury-induced immunodepression” has been proposed to describe this immune deficiency syndrome [105]. Although direct evidence is still lacking, prolonged immunodepression following TBI or stroke may impair the ability of immune cells to eliminate senescent cells, leading to an accumulation of these cells in the brain (Fig. 1).

Apart from the immune cell-mediated clearance pathway, the glymphatic system/meningeal lymphatic system, a waste clearance pathway in the brain, is also proposed to be involved in the clearance of senescent cells [112]. First identified in 2012, the glymphatic system is reported to clear metabolites and soluble proteins such as Aβ [113, 114]. Since its discovery, this system has received increasing attention from the scientific community, with research expanding to various neurological disorders. Enhancing glymphatic lymphatic drainage has been shown to reduce Aβ deposition in the brain, and mitigate learning and memory deficits in transgenic mouse models of AD [115]. Importantly, impaired glymphatic system function has been widely observed in TBI and stroke [116–119]. Severe deficits in glymphatic drainage were detected in an experimental mouse model of TBI, occurring within hours and lasting for months [116]. Pre-existing glymphatic dysfunction can exacerbate cognitive deficits after TBI, while enhanced glymphatic drainage has been found to prevent TBI-induced gliosis [116]. Compromised glymphatic system perfusion has been reported in four different animal models of stroke, as evidenced by reduced brain clearance of low-molecular-weight compounds [118]. Similarly, a neuroimaging study revealed disrupted glymphatic function in patients with ischemic stroke when compared to age-matched healthy individuals [120]. Moreover, impaired glymphatic drainage may worsen the accumulation of Aβ in the brain [116, 121]. This insufficient Aβ efflux, resulting from dysfunctional glymphatic function after TBI/stroke, may further trigger the formation of senescent cells. Consequently, glymphatic dysfunction is increasingly recognized as a major contributor to brain injury-induced neurodegeneration [122].

A recent study has uncovered a novel function of the glymphatic system: facilitating the removal of senescent cells from the brain [112]. Initially, researchers observed senescent astrocytes in perivascular spaces in both aged mice and brains of elderly individuals [112]. Subsequent investigations revealed that blocking the glymphatic drainage pathway increases the accumulation of senescent perivascular astrocytes, whereas enhancing glymphatic drainage significantly reduces their numbers in aged mice [112]. These findings shed new light on the role of this waste clearance system in eliminating senescent cells and suggest the hypothesis that impaired glymphatic system function might exacerbate the accumulation of senescent cells in the brain following TBI and stroke (Fig. 1). However, further studies are needed to verify this hypothesis.

## Senescence in different cell types after brain injury

**Fig. 2:**
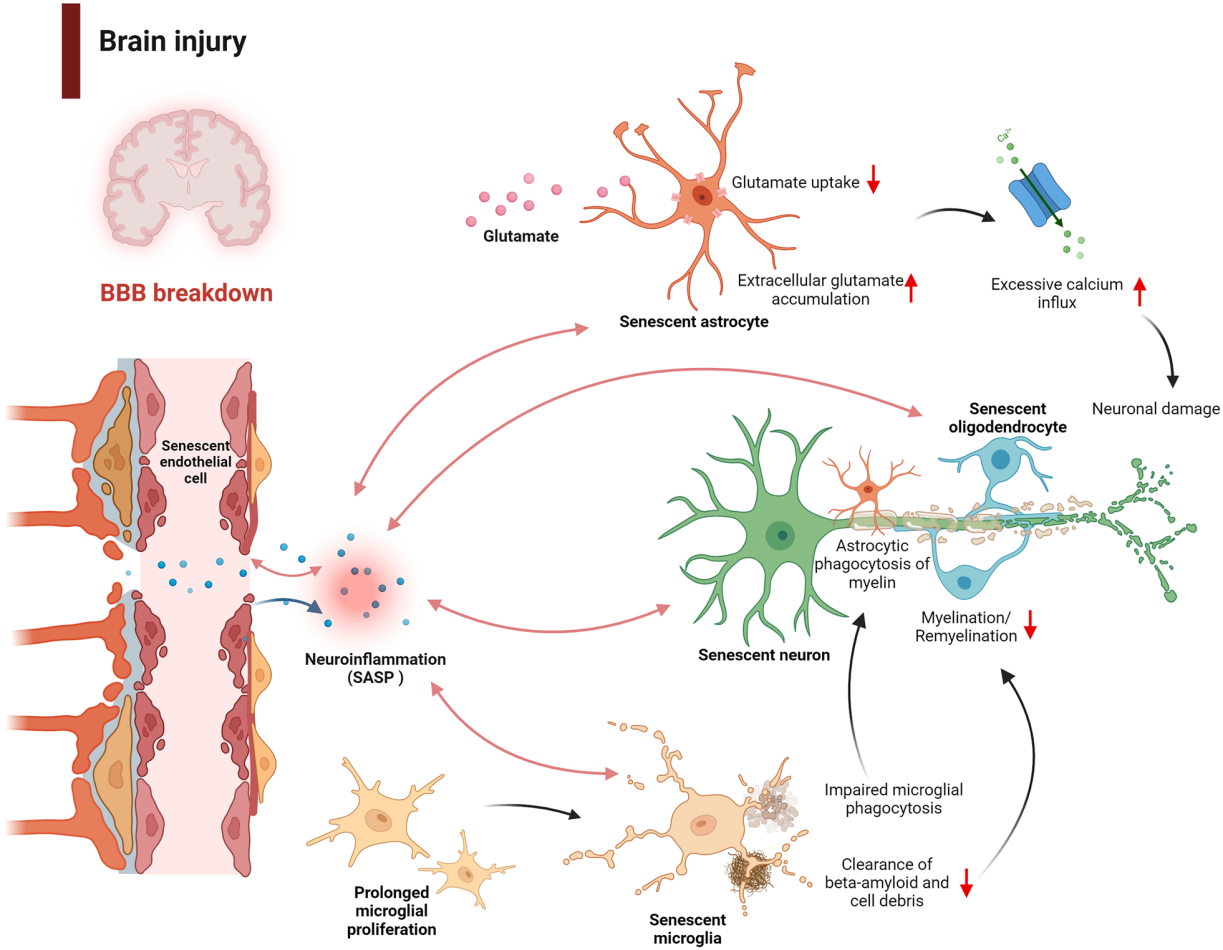
Consequences of senescence in different cell types and their potential interplay. Breakdown of the BBB following brain injury can lead to the infiltration of cytotoxic mediators and immune cells into the brain parenchyma, creating a proinflammatory environment that perpetuates cellular senescence. Senescence in endothelial cells can further compromise the BBB, creating a negative feedback loop that exacerbates the damage. Prolonged microglial proliferation may promote senescence within themselves, reducing their ability to phagocytose cellular debris and beta-amyloid, thereby hindering remyelination after brain injury. The compromised phagocytosis by microglia may also induce astrocytes to phagocytose myelin, potentially worsening demyelination. Senescent astrocytes may have diminished glutamate transport capacity, resulting in reduced glutamate uptake. This leads to the accumulation of extracellular glutamate, causing excessive calcium influx and, ultimately, neuronal damage. Oligodendrocyte senescence, triggered by proinflammatory signals, can inhibit their maturation into myelinating oligodendrocytes, leading to insufficient myelination and possible axonal degeneration. Inflammation may be a mediator underlying the communications between different cells. Senescent cells not only exacerbate inflammation but may also be induced by a proinflammatory microenvironment. Abbreviations: BBB, Blood–brain barrier

Evidence has shown that senescent cells can significantly accumulate in the CNS following brain injury. Despite this, the cell types most susceptible to senescence under these pathological conditions are not well understood. In the following section, we discuss the potential consequences of senescence in different cell types and their interactions (Fig. 2). Further investigations are required to elucidate specific cell types that undergo senescence following brain injury. These insights may pave the way for targeted therapeutic strategies.

### Neurons

Neurons, traditionally considered postmitotic cells, were once thought incapable of becoming senescent due to their terminally differentiated nature. However, recent evidence suggests that neurons can acquire a senescent phenotype under certain pathological conditions [123–125], although the exact mechanisms remain largely unknown. A postmortem study using a senescence eigengene approach profiled senescent cells in patients with AD and found that over 97% of the observed senescent cells were excitatory neurons [70]. These neurons also overlapped with tau-containing neurofibrillary tangles [70]. In aged mice, markers such as DNA damage, activated p38 MAP kinase, oxidative damage, and high SA-β-Gal activity have been reported in cortical, hippocampal, and peripheral neurons, accounting for 20%–40% of the total neuronal population [123]. Single-cell RNA sequencing has revealed a senescence-like expression profile in neuronal clusters in TBI mouse brains. These neuronal clusters are characterized by DNA damage, senescence-associated inflammation, and senescence-associated anti-apoptotic phenotypes [90]. This provides further evidence that brain injury may also induce senescence in neurons. Despite these insights, further research is needed to elucidate the mechanisms driving neuronal senescence and its potential interactions with other cell types.

### Microglia

As the resident innate immune cell, microglia are the primary phagocytic cells in the brain, playing a critical role in maintaining brain homeostasis [126]. Microglia typically become activated in response to infection or injury to eliminate pathogenic molecules and cellular debris. The activation and proliferation of microglia are common pathological features in TBI and stroke [127, 128]. However, excessive microglial proliferation can contribute to neuronal deterioration. Prolonged microglial proliferation has been reported to promote replicative senescence in microglia [129]. Inhibition of early microglial proliferation, in contrast, prevents the onset of microglial senescence in mouse models of neurodegeneration and amyloidosis, accompanied by mitigated amyloid-related pathology and synaptic dysfunction [129]. A possible explanation for these findings is that microglial senescence might compromise its phagocytic function, as microglia are responsible for the physiological clearance of Aβ and cellular debris [130–132]. Preventing microglial senescence can potentially restore their phagocytic activity, thereby counteracting amyloid-related pathology. Indeed, the phagocytic activity of microglia is essential for maintaining brain homeostasis. Upon injury, microglia are recruited to the lesion site for debris clearance, and pharmacological or genetic inhibition of their phagocytic action augments secondary cell death after TBI [132].

Given the vital role of microglia in maintaining myelin integrity and proper myelination, microglial senescence may also lead to myelin dysfunction. Through the phagocytosis of myelin debris, microglia contribute to remyelination following demyelinating insults [133]. Remyelination is impeded when microglial phagocytic activity is impaired [133, 134]. Recent evidence shows that although microglia do not directly contribute to oligodendrocyte maturation and myelination, they also play a critical role in the growth and maintenance of myelin health in adulthood [135]. The deletion of microglia leads to myelin dysfunction and impaired cognitive performance in mice, which is believed to be linked to a disrupted TGFβ1–TGFβR1 signaling axis between microglia and oligodendrocytes [135]. Inadequate Aβ clearance may also contribute to myelin breakdown. Although soluble Aβ treatment does not impact myelinogenesis in either in vivo or in vitro models, demyelination is evident around Aβ plaques in amyloidosis transgenic mice [136]. This suggests that inadequate microglial clearance of Aβ due to microglial senescence may accelerate Aβ plaque accumulation and further contribute to myelin breakdown. Senescence in microglia, therefore, could disrupt various physiological processes crucial for proper myelin function. However, whether myelin dysfunction also plays a role in driving microglial senescence is still being explored.

Together, microglial senescence could compromise its phagocytic function for cellular debris and Aβ, and disrupt signaling pathways crucial for effective myelination/remyelination. The interaction of these processes could collectively contribute to neuronal degeneration. The crosstalk between microglia and other cell types is also intriguing and will be discussed in the following sections.

### Astrocytes

Astrocytes, the most abundant glial cells in the CNS, play critical roles in regulating inflammation, ionic homeostasis, neuronal support, and neurovascular functions [137, 138]. Senescent astrocytes have been thought to contribute to age-related tissue deterioration [139, 140]. Senescent astrocytes, featured by loss of lamin-B1, were observed in the hippocampus of aged mice and the post-mortem brains of non-demented elderly humans [86]. Similarly, transgenic mice with tauopathy exhibit widespread senescent astrocytes and microglia [74]. Removing these senescent cells through genetic or pharmacological methods has been shown to rescue cortical and hippocampal neurons from degeneration and improve cognitive function [74]. Moreover, protein expression arrays reveal that senescent astrocytes in culture produce increased levels of inflammatory cytokines [140], which may drive neuronal degeneration. This suggests that astrocytic senescence is linked to both inflammation and neurodegeneration.

Notably, astrocytic senescence may also disrupt glutamate homeostasis. As an essential neurotransmitter, glutamate is crucial for synaptic plasticity. Excess extracellular glutamate, nevertheless, can be neurotoxic. Astrocytes typically help prevent glutamate excitotoxicity by taking up excess glutamate from the extracellular space [141]. Evidence shows that senescent primary human astrocytes, induced by X-irradiation, exhibit reduced expression of glutamate transporters [142]. This downregulation makes neurons more vulnerable to glutamate toxicity. In pure neuronal cultures, exposure to 10 mM glutamate results in significant cell death [142]. Interestingly, when neurons are co-cultured with healthy astrocytes in the same glutamate concentration, neither cell type dies. However, significant neuronal death occurs when neurons are co-cultured with senescent astrocytes under similar conditions [142]. Therefore, the impaired glutamate uptake by senescent astrocytes can potentially exacerbate glutamate excitotoxicity, ultimately leading to neuronal cell death. As a shared pathological event, glutamate dyshomeostasis is frequently observed in both preclinical models and patients with TBI and stroke [143–145]. This observation raises the possibility that senescence in astrocytes may worsen neuronal damage following brain injury due to impaired glutamate uptake.

The potential interplay between astrocytes and microglia is also noteworthy. Research has shown that impaired microglial phagocytosis can lead to compensatory phagocytic activity by astrocytes [146]. This compensatory mechanism, however, could be detrimental, as it has been associated with demyelination following CNS insults [147, 148]. In this sense, exploring the communication between senescent signaling in microglia and astrocytes could provide valuable insights into the development of therapeutic compounds.

### Oligodendrocytes

Oligodendrocytes, the myelinating cells in the brain, originate from oligodendrocyte progenitor cells (OPCs). Well-functioning myelin is essential for axon integrity and the preservation of cognition [149, 150]. Myelin dysfunction has been implicated in various neurodegenerative diseases [151–153]. Although the connection between brain injury and senescence in oligodendrocytes or OPCs has not been well studied, evidence indicates that oligodendrocytes can undergo senescence under certain conditions [154, 155]. Comparison of directly converted oligodendrocytes from young, adult, and old human donors shows a decrease in the proportion of mature myelinating oligodendrocytes with increasing donor age [155]. This reduced myelination capacity is associated with increased ROS production and upregulation of cellular senescence signaling, suggesting that aging-related signals may trigger oligodendrocyte senescence and impair myelination [155]. Pro-inflammatory cytokines from other glial cells can also induce senescence in oligodendrocytes. Exposure to supernatants from pro-inflammatory microglia elevates senescence markers and impedes the differentiation of oligodendrocytes from young human donors [155]. Of note, the NF-κB pathway has been identified as a critical mediator of post-mitotic senescence in oligodendrocytes. Transgenic mice with persistent NF-κB activation in mature myelinating oligodendrocytes exhibit significant myelination deficits and white matter loss [154]. RNA-Seq analysis further reveals that primary oligodendrocytes isolated from these mice show elevated SA-β-Gal activity and SASP gene expression profiles [154]. More importantly, oligodendrocytes from TBI mouse brains also display senescence markers and upregulation of NF-κB pathway-associated genes [154]. This study provides the first evidence that CNS insult could trigger senescence in oligodendrocytes, highlighting the potential of targeting the NF-κB pathway to mitigate oligodendrocyte senescence and its detrimental effects on axonal myelination and neuronal function.

Collectively, oligodendrocyte senescence, whether induced by pro-inflammatory signals from other glial cells or directly by brain injury, may impair axonal myelination and lead to neuronal deterioration.

### Endothelial cells

Endothelial cells, the major component of the blood–brain barrier (BBB), are crucial for maintaining its integrity, which prevents the infiltration of neurotoxic plasma components and pathogens into the brain [156, 157]. The existence of this barrier helps preserve homeostasis within the brain. It is noteworthy that senescence in endothelial cells can directly impair BBB integrity [158]. In an in vitro model of the BBB, the use of senescent endothelial cells and pericytes led to disrupted tight junction structures and compromised barrier integrity [158]. Interestingly, the removal of these senescent endothelial cells can rescue the reduced microvascular density and increased BBB permeability observed after chronic whole-brain irradiation [159]. Indeed, BBB breakdown has been implicated in prolonged neuroinflammation [160, 161]. This allows the infiltration of cytotoxic mediators, which may contribute to a proinflammatory microenvironment within the brain. Dysfunctional BBB is also commonly observed in TBI and stroke, and this disruption can persist for months after the initial insult [162, 163]. Elevated proinflammatory signals resulting from BBB breakdown could potentially induce senescence in various cell types. Senescent endothelial cells, in turn, might exacerbate BBB disruption, leading to a detrimental feedback loop. Nevertheless, further research is needed to clarify the interactions between senescent endothelial cells and other cell types within the CNS.

## Discovery of senolytic compounds

The first senolytic, identified by Zhu and colleagues, was Dasatinib (D) + Quercetin (Q), a drug cocktail shown to selectively clear senescent cells by inhibiting pro-survival pathways/antiapoptotic pathways within these cells [164]. Since then, an increasing number of senolytic compounds have been identified, including various BCL-2 family inhibitors, p53 modulators, heat shock protein 90 (HSP90) inhibitors, natural products, cardiac glycosides, and biguanides (Table 1).

**Table 1:** Summary of identified senolytic compounds and associated clinical trials

Compound	Class	Senolytic clinical trial
Dasatinib + Quercetin [164]	BCL-2 family inhibitors	Patients with IPF: NCT02874989; Patients with CKD: NCT02848131
		Patients with schizophrenia and TRD: NCT05838560
		Patients with AD: NCT04063124, NCT04785300
		Patients with MCI or AD: NCT04685590
		Patients with NAFLD: NCT05506488
Navitoclax (ABT263) [165]	BCL-2 family inhibitor	N/A
ABT737 [166]	BCL-2 family inhibitor	N/A
A1331852 [167]	BCL-2 family inhibitor	N/A
A1155463 [167]	BCL-2 family inhibitor	N/A
UBX1967 [168]	BCL-2 family inhibitor	N/A
Foselutoclax (UBX1325) [169]	BCL-2 family inhibitor	Patients with diabetic macular edema and age-related macular degeneration: NCT04537884
		Patients with diabetic macular edema: NCT04857996
FOXO4-DRI [170]	p53 modulator	N/A
UBX0101 [171]	p53 modulator	Patients with osteoarthritis: NCT03513016, NCT04349956, NCT04229225, NCT04129944
Alvespimycin (17-DMAG) [172]	HSP90 inhibitor	N/A
Geldanamycin [172]	HSP90 inhibitor	N/A
Tanespimycin (17-AAG) [172]	HSP90 inhibitor	N/A
XL888 [173]	HSP90 inhibitor	N/A
Fisetin [174]	Natural product	Healthy individuals and older patients with multiple chronic diseases: NCT06431932
		Patients with COVID-19: NCT04771611, NCT04537299, NCT04476953
		Patients with osteoarthritis: NCT04210986, NCT04770064
		Patients with peripheral artery disease: NCT06399809
		Vascular function in older adults: NCT06133634
Curcumin [178]	Natural product	N/A
Piperlongumine [175]	Natural product	N/A
Luteolin [176]	Natural product	N/A
Procyanidin C1 [177]	Natural product	N/A
EF24 [179]	Curcumin analog	N/A
Ouabain [180]	Cardiac glycoside	N/A
Proscillaridin A [181]	Cardiac glycoside	N/A
Digoxin [181]	Cardiac glycoside	N/A
Metformin [182]	Biguanide	Aging-related indicators in middle-aged and elderly people: NCT06459310
		Muscle regrowth in older adults: NCT06185179

*IPF*, Idiopathic pulmonary fibrosis; *CKD*, Chronic kidney disease; *MCI*, Mild cognitive impairment; *TRD*, Treatment-resistant depression; *NAFLD*, Non-alcoholic fatty liver disease; CLL, Chronic lymphocytic leukemia; *N/A*, Not applicable; *HSP90*, Heat shock protein 90

Numerous BCL-2 inhibitors have emerged as promising senolytics. Among these, navitoclax (ABT263), a first-generation senolytic and specific inhibitor of the anti-apoptotic proteins BCL-2 and BCL-xL, has garnered considerable attention [165]. This class of drugs disrupt senescent cell anti-apoptotic pathways, leading to the selective induction of apoptosis in senescent cells [165–169]. Targeted modulation of p53-associated signaling has also demonstrated senolytic potential. Disrupting the interaction between the transcription factor FOXO4 and p53 using FOXO4-DRI, a designed peptide, selectively induces p53 nuclear exclusion and intrinsic apoptosis in senescent cells [170]. Similarly, UBX0101, a p53-destabilizing MDM2 inhibitor, interferes with the MDM2-p53 interaction, resulting in the apoptosis of senescent cells [171]. Since HSP90, a molecular chaperone, plays a role in the anti-apoptotic mechanisms, several HSP90 inhibitors have been investigated for their senolytic potential [172, 173]. These studies revealed that HSP90 inhibitors disrupt the HSP90-AKT interaction, destabilizing the active, phosphorylated form of AKT, which in turn induces apoptosis in senescent cells [172].

Several natural products, such as fisetin [174], piperlongumine [175], luteolin [176], procyanidin C1 [177], curcumin [178], and its analogs [179], have also been identified as senolytics, acting through various mechanisms. In addition, certain cardiac glycosides also exhibit senolytic activity [180, 181]. Potential mechanisms include the inhibition of Na^+^/K^+^-ATPase, resulting in plasma membrane depolarization in senescent cells and subsequent acidification, or the activation of the pro-apoptotic BCL2 family member NOXA, which triggers apoptosis in these cells. Intriguingly, the antidiabetic drug metformin has also been repurposed as a senolytic compound, although the exact mechanisms underlying its senolytic action remain incompletely understood [182, 183]. Several of these senolytic compounds have advanced to clinical trials, with encouraging outcomes. Numerous large randomized controlled trials are either in progress or planned, as outlined in Table 1.

Apart from the compounds discussed above, emerging senotherapeutics are being identified. For example, multiple lines of evidence indicate that the programmed death-ligand 1 (PD-L1), an immune checkpoint molecule, is heterogeneously expressed in senescent cells, potentially facilitating their immune escape [184, 185]. Further investigation revealed that blocking the PD1/PDL1 immune checkpoint significantly reduces the number of senescent cells in naturally aging mice through the activation of CD8^+^ T cells [185]. As a therapeutic target in cancer, the PD1/PDL1 immune checkpoint has attracted considerable research interest. Several PD-L1 monoclonal antibodies have shown promising effects in clinical settings [186]. Repurposing anti-PD1/PDL1 therapy as a senotherapy could be an appealing field of study, although it remains to be fully verified. Indeed, high-throughput screening and machine learning are now being utilized to identify emerging senolytics [173, 187]. Through these efforts, it is anticipated that suitable senotherapeutics will be discovered for patients with a range of medical conditions.

## Senolytic therapies for TBI and stroke: insight from experimental animals

Several studies have explored the efficacy of senolytic therapies for TBI. In a mouse model of mild closed TBI, a single dose of the senolytic agent ABT263, administered one-week post-injury, reduced the number of senescent cells in the brain, despite the persistence of DNA damage and pro-inflammatory signaling [90]. Furthermore, mice treated with ABT263 exhibited enhanced learning and memory performance two weeks post-injury compared to those treated with vehicles [90]. This suggests that eliminating senescent cells in the brain during the acute/subacute phase may lead to more favorable outcomes.

Similarly, another study investigated the therapeutic potential of the D + Q drug cocktail in TBI [22]. Administered one-month post-injury for 13 weeks (three consecutive days per week), this drug combination significantly reduced senescent cells in the brain, accompanied by mitigated neurodegeneration, neuronal loss, and improved cognitive functions [22]. Consequently, the elimination of persistent senescent cells following TBI may offer protection against TBI-associated neurodegeneration.

Several studies have reported the therapeutic benefits of senolytic therapy in experimental animal models of cerebral ischemia [96, 100]. The systemic administration of ABT263 started 24 h after MCAO, effectively reducing infarct volume and improving neurological function in rats [100]. Moreover, the local elimination of senescent cells in the peri-infarct area using the lenti-INK-ATTAC viral vector also resulted in better motor and overall neurological functions [96].

Interestingly, before its identification as a senolytic compound, the flavonoid fisetin was recognized for its neuroprotective properties [188]. Administered either before or after MCAO, fisetin reduces TNFα production and decreases infarct size [188]. Its neuroprotective effects have also been demonstrated in TBI models [189], although these studies did not directly assess the burden of senescent cells.

**Fig. 3:**
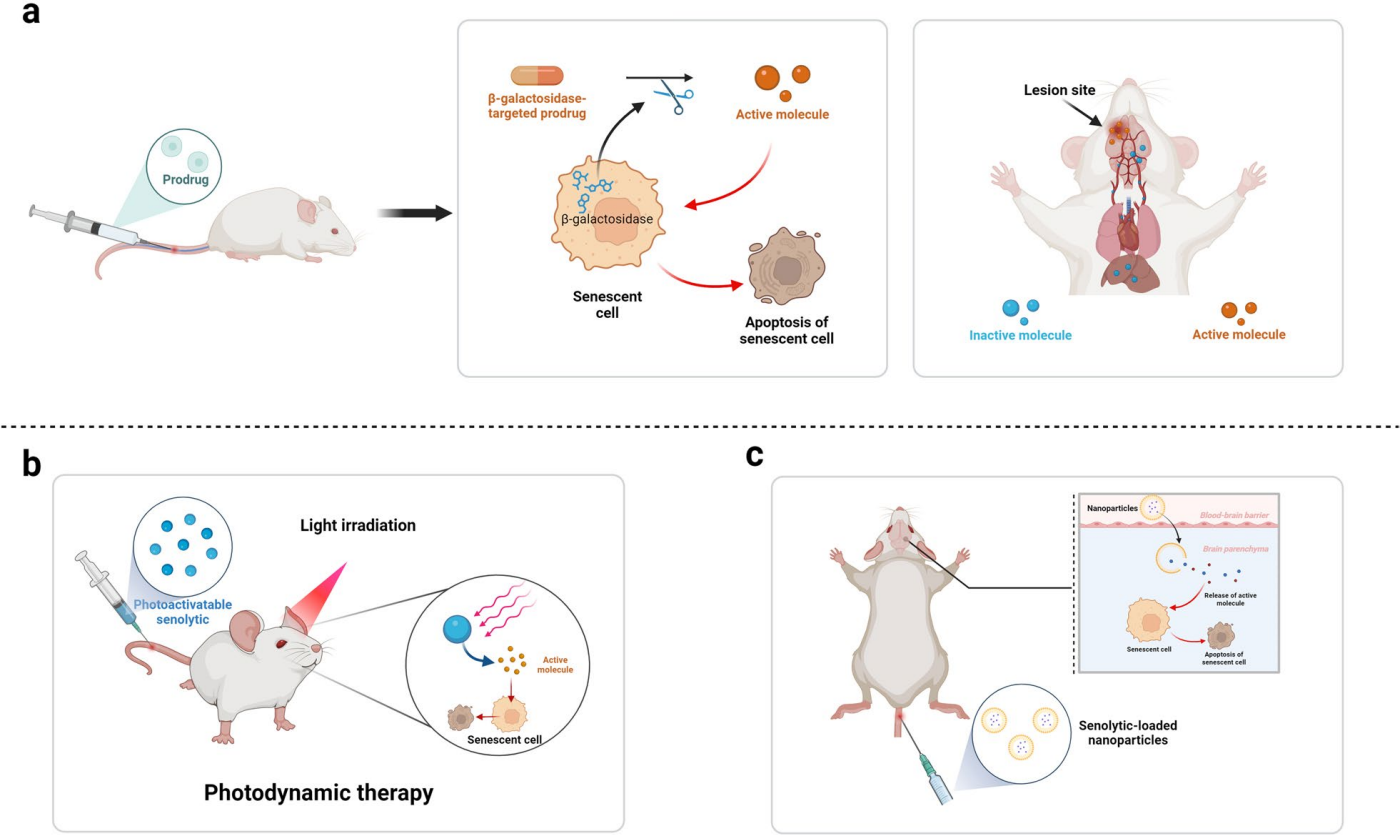
Summary of potential approaches to enhance the therapeutic targeting and efficacy of senolytic agents. **a** A diagram showing the mechanism of action of a β-galactosidase-targeted senolytic prodrug. After administration, the prodrug is cleaved into active molecules by β-galactosidase expressed in senescent cells. This allows the senolytic to specifically target these cells and induce their apoptosis. **b** Summary of the potential application of photodynamic therapy with senolytics. After administration, the photoactivatable senolytics are activated by light, leading to the release of active molecules in the targeted tissue. **c** Summary of the potential application of combining nanoparticles with senolytics. Utilizing desired nanoparticles, the drug molecules may more effectively cross the blood–brain barrier, resulting in higher bioavailability

Overall, although research in this field is still in the preliminary stage, these findings provide valuable insights into the therapeutic potential of senolytic therapies for TBI and stroke (Fig. 3).

## Senolytic therapies for TBI and stroke: from bench to bedside

**Fig. 4:**
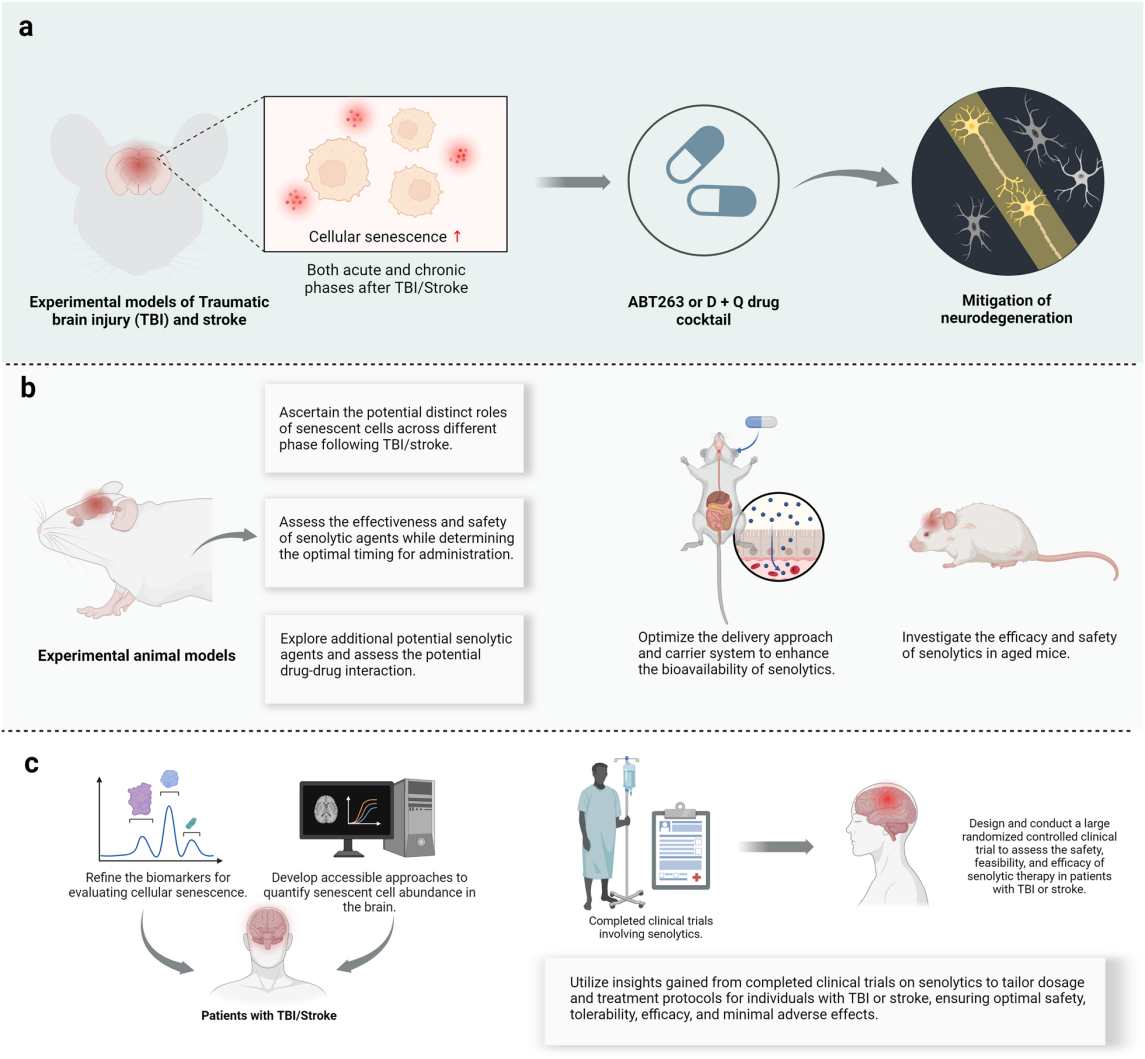
Summary of experimental evidence supporting the therapeutic value of senolytic therapy for TBI/stroke and directions for future research. **a** Experimental evidence indicates that cellular senescence occurs during both acute and chronic phases following TBI/stroke. Senolytics such as ABT263 and the D + Q drug cocktail, administered during the subacute or chronic phases post-TBI/stroke, demonstrate efficacy in mitigating secondary neurodegeneration. **b** Summary of future directions for research on senolytic therapy for TBI/stroke. **c** Clinical translation of senolytic therapy for TBI/stroke. Abbreviations: TBI, Traumatic brain injury; D, Dasatinib; Q, Quercetin

While the experimental evidence on the therapeutic potential of senolytic therapy for TBI and stroke is promising, several key challenges must be addressed before it can be applied clinically. Comprehensive preclinical research and rigorous clinical trials will be essential for successfully translating senolytic therapies from the lab to clinical practice (Fig. 4).

### Therapeutic window

First, cellular senescence following brain injury may be a progressive pathological process that develops over time. Indeed, a high level of senescent cells has been observed one year after TBI [88]. Hence, it is crucial to determine to what extent these senolytic agents can clear senescent cells post-TBI and how long their anti-senescence effect lasts, as inadequate clearance may lead to a “relapse” of senescence. It is also important to note that the incidence and prevalence of TBI/stroke are higher among the elderly. A significant proportion of TBI cases are fall-related and typically occur in older adults [190]. Studies have shown that aged animals have limited endogenous brain repair capabilities after TBI or stroke compared to younger animals [191, 192]. Despite this, most preclinical studies use young animals, which may not accurately represent the physiological responses of older populations to brain injury. Therefore, further research is warranted to evaluate the efficacy of senolytic therapies in aged animals and to explore potential differences in outcomes between young and aged animals. This could provide valuable insights into the therapeutic potential of senolytics for elderly individuals with TBI or stroke.

Second, as discussed earlier, cellular senescence may play different roles after brain injury, with early senescence potentially being beneficial while prolonged senescence may be detrimental. Thus, determining the optimal treatment window would provide valuable insights into clinical practice. Additionally, research and clinical practice have not adequately focused on the progression of secondary injury following TBI/stroke. As a result, most patients may miss the optimal treatment window for senolytic therapies. It is essential to explore whether the effectiveness of senolytic agents can extend to longer windows. This would provide valuable insights into the potential applicability of this strategy for diverse patient populations.

### Emerging senotherapies: therapeutic promise and potential drug interactions

As discussed in previous sections, numerous senolytic compounds have been identified, acting through different mechanisms of action. Nevertheless, available studies have primarily evaluated the therapeutic value of first-generation senolytics, such as the D + Q drug combination and ABT263, in TBI and stroke. In this regard, the potential benefits of other emerging senolytics also deserve evaluation, which may provide more diverse and optimal senotherapy options for patients with various medical conditions. Given the potentially impaired clearance of senescent cells following brain injury, a combined therapeutic approach may prove more effective. For example, strategies that both promote the demise of senescent cells and enhance the capacity of the glymphatic system to remove these cells could offer a promising treatment option.

Moreover, current knowledge of the potential side effects associated with available senolytics remains limited. For instance, high doses of some BCL-2 family inhibitors may inadvertently inhibit anti-apoptotic signaling in other cell types, potentially causing transient thrombocytopathy [193]. In advanced atherosclerotic mice, treatment with ABT263 leads to a significant decrease in smooth muscle cell numbers, accompanied by increased mortality [194]. Therefore, further studies are warranted to assess the potential toxic or adverse effects of long-term dosing.

Investigating potential drug-drug interactions with senolytic therapy is also crucial for its safe clinical application. Clinically, patients with TBI or stroke typically need to take specific medications to enhance functional outcomes. However, little is known about the potential interactions between senolytic drugs and these medications for patients. Comprehensive preclinical research and rigorous clinical trials are therefore vital to ensure that senolytic therapy can be safely integrated into existing treatment protocols without compromising patient outcomes.

### Bioavailability of senolytic compounds

Most senolytic agents are typically administered systemically, although this may not be the most optimal route for treating brain injury. Due to the presence of the BBB, many drugs cannot effectively reach their targets within the brain, leading to limited bioavailability. In a clinical trial involving the D + Q drug cocktail, for instance, oral administration led to increased levels of both D and Q in the blood of all participants [195]. However, only elevated levels of D, not Q, were detected in the CSF [195]. Therefore, successful treatment necessitates the development of delivery methods capable of facilitating drug passage across the BBB. In this regard, nanotechnology may present a promising approach. This approach allows the transportation of medications in the form of nanoparticles across biological barriers, thereby overcoming such limitations [196].

Another challenge with senolytic therapies is the significant heterogeneity in the accuracy and broad-spectrum activity of many senolytic drugs [197]. This variability makes it difficult to ensure their effectiveness against different types of senescent cells while also limiting their potential toxicity to non-senescent cells. A potential solution to this issue is the development of prodrug strategies that specifically target senescent cells [198, 199]. For instance, a prodrug targeted by β-gal has been reported to selectively deplete senescent cells across a wide spectrum of cell types and tissues [199]. As a lysosomal hydrolytic enzyme, β-gal shows increased activity in senescent cells, making it a reliable biomarker for cellular senescence [78]. Activated specifically by β-gal, the prodrug SSK1 is cleaved into cytotoxic gemcitabine, which induces apoptosis in senescent cells [199]. Studies have shown that this compound effectively reduces senescent cell burden, diminishes the SASP, lowers low-grade local and systemic inflammation, and improves physical function in aged mice [199]. Also, the combination of senolytics with photodynamic therapy, which involves the use of light and a photosensitizing drug to target specific cells or tissues, may further improve the targeting and efficacy of the therapy. To limit the off-target effects of these small-molecule drugs, a photosensitive β-gal-targeted senolytic prodrug has been developed [200]. This prodrug can be selectively activated by β-gal in response to light illumination, ensuring higher tractability, broad-spectrum activity, and accuracy [200]. These emerging strategies are expected to propel senolytic drugs into clinical testing (Fig. 3).

Monitoring senescent cell burden and therapeutic response is also vital, and reliable biomarkers would aid in tracking treatment efficacy. Although several biomarkers for senescence have been proposed, as described in previous sections, there is no refined approach to measuring senescent cells in the brain. Indeed, the evaluation of senescent cell burden largely relies on the analysis of SASP factors or senescence markers in body fluids, which only permits the assessment of systemic senescence burden. The development of accessible and reliable approaches to quantify senescent cell abundance in the brain, in this context, might further facilitate the application of senolytic therapies in clinical settings. These approaches would enable direct measurement of the progression of cellular senescence in the brain and evaluation of the effectiveness of senolytic therapies. One potential avenue for achieving this goal is through introducing a nanoparticle-based theranostic system, which allows precise diagnosis and real-time monitoring of treatment response through synergetic multimodal imaging [201, 202]. Such advancements could also pave the way for future clinical trials of senolytic agents in TBI and stroke, as well as other neurological disorders.

### Gap between laboratory experiments and clinical practice

Recognizing the physiological and metabolic differences between experimental animals and humans is vital, as these discrepancies often limit the direct comparability of animal experiment results to human outcomes. To enhance clinical translation, efforts must focus on narrowing the gap between preclinical and clinical research. In this regard, the use of non-human primates might help bridge this gap by better simulating human physiology and disease, thereby enhancing the translation of laboratory findings into clinical practice. Additionally, the dosages of senolytics used in experimental animals often exceed the clinically recommended levels for humans, and the half-life of each senolytic compound varies significantly [203]. Consequently, the optimal dosage and administration strategy—whether continuous or intermittent—still require validation through larger clinical trials.

Despite these challenges, preliminary clinical trials have highlighted the promising potential of senolytic therapies in treating human diseases, including neurodegenerative disorders [195, 204]. For example, a pilot clinical trial [195] demonstrated the safety, tolerability, and feasibility of D + Q senolytic therapy in AD patients. Additionally, ongoing clinical trials (Table 1) investigating various senolytic compounds are expected to provide valuable insights into their applicability for patients with TBI and stroke. With these concerted efforts, the clinical translation of senolytic therapy for TBI and stroke is eagerly anticipated.

## Conclusion

The devastating consequences of brain injury, particularly the increased risk of neurodegeneration, are increasingly acknowledged. This review offers critical insights into the role of cellular senescence in secondary neurodegeneration following TBI and stroke. A growing body of evidence underscores a strong connection between cellular senescence, inflammation, and neurodegeneration. Notably, senescent cells, a common pathological feature, are present in the brain after TBI or stroke. Although the precise vulnerability of different cell types to senescence and their interactions remain underexplored, the targeted elimination of these cells has yielded promising preliminary results in mitigating brain injury-induced neuronal degeneration. These findings highlight a novel therapeutic target for addressing secondary neurodegeneration following brain insult. From a translational standpoint, further rigorous investigation into the safety and efficacy of senolytic agents is imperative, as it holds the potential to open new avenues for managing the long-term consequences of brain injury.

## Abbreviations

AD: Alzheimer’s disease
Aβ: Amyloid-beta
CTE: Chronic traumatic encephalopathy
CNS: Central nervous system
CSF: Cerebrospinal fluid
β-gal: β-Galactosidase
SA-β-Gal: Senescence-associated β-galactosidase
HSP90: Heat shock protein 90
TBI: Traumatic brain injury
BBB: Blood–brain barrier
MCAO: Middle cerebral artery occlusion
ROS: Reactive oxygen species
OPCs: Oligodendrocyte progenitor cells
SASP: Senescence-associated secretory phenotype
D: Dasatinib
Q: Quercetin
PD: Parkinson’s disease
PD-L1: Programmed death-ligand 1
